# A One Health Approach to Investigating *Leptospira* Serogroups and Their Spatial Distributions among Humans and Animals in Rio Grande do Sul, Brazil, 2013–2015

**DOI:** 10.3390/tropicalmed4010042

**Published:** 2019-02-27

**Authors:** Noemi Polo, Gustavo Machado, Rogerio Rodrigues, Patricia Nájera Hamrick, Claudia Munoz-Zanzi, Martha Maria Pereira, Marilina Bercini, Loeci Natalina Timm, Maria Cristina Schneider

**Affiliations:** 1 Pan American Health Organization (PAHO/WHO), Washington, DC 20037, USA; najerapa@paho.org; 2 Department of Population Health and Pathobiology, College of Veterinary Medicine, North Carolina State University, Raleigh, NC 27606, USA; gmachad@ncsu.edu; 3 Instituto de Pesquisas Veterinárias Desidério Finamor (IPVDF), Secretaria de Agricultura do Rio Grande do Sul, Eldorado do Sul 92990-000, Brazil; rogerrodriguesvet@gmail.com; 4 Division of Environmental Health Sciences, School of Public Health, University of Minnesota, Minneapolis, MN 55455, USA; munozzan@umn.edu; 5 Instituto Oswaldo Cruz, WHO Collaborating Center for Leptospirosis, Rio de Janeiro, Rio de Janeiro 21040-900, Brazil; mpereira@ioc.fiocruz.br; 6 Centro Estadual de Vigilancia Ambiental, Secretaria de Saude do Rio Grande do Sul, Porto Alegre 90610-030, Brazil; mariber1954@gmail.com; 7 Centro Estadual de Vigilancia de Saude, Laboratorio Central do Estado Rio Grande do Sul, Porto Alegre 90450-190, Brazil; loeci-timm@saude.rs.gov.br

**Keywords:** leptospirosis, serogroups, One Health, zoonoses, global health, infectious disease, Brazil

## Abstract

Leptospirosis is an endemic zoonotic disease in Brazil and is widespread throughout rural populations in the state of Rio Grande do Sul. This study aimed to identify presumptive infecting *Leptospira* serogroups in human and animal cases and describe their occurrences within the ecoregions of the state by animal species. Data for human and animal leptospirosis cases were gathered from the government’s passive surveillance systems and presumptive infecting serogroups were identified based on a two-fold titer difference in serogroups in the microscopic agglutination test (MAT) panel. A total of 22 different serogroups were reported across both human and animal cases. Serogroup Icterohaemorrhagiae was the most common among humans, while serogroup Sejroe predominated among animal cases, particularly bovines. Each ecoregion had a large distribution of cases, with 51% of the human cases in the Parana–Paraiba ecoregion, and 81% of the animal cases in the Savannah ecoregion. Identifying and mapping the serogroups circulating using the One Health approach is the first step for further understanding the distribution of the disease in the state. This study has the potential to aid in guiding public health and agricultural practices, furthering the need for a human vaccine in high-risk populations to complement control and prevention efforts.

## 1. Introduction 

Leptospirosis, a zoonotic bacterial disease, is one of the most important neglected tropical bacterial diseases in Latin America and the Caribbean and is among the leading zoonotic causes of morbidity worldwide [1]. This zoonotic disease has a higher incidence in sub-tropical and impoverished populations in developing countries. It primarily affects vulnerable populations, with an estimated annual global incidence of 1.03 million people and 58,900 deaths [2,3]. Even so, this number is assumed to be an underestimate, as leptospirosis is commonly misdiagnosed with other febrile illnesses such as dengue, malaria, and chikungunya, and therefore is likely underreported [2]; especially within rural populations where access to health facilities is limited and awareness of the disease is low. 

Brazil is the country with the highest number of reported annual cases of leptospirosis in the region of the Americas, with an average of 3890 cases annually and an approximate 10% fatality rate [4,5]. The state of Rio Grande do Sul (RS), specifically, has the fifth highest number of cases of leptospirosis in the country, with an average of 429 cases a year [6,7]. According to Schneider et al. 2015 [6], the risk for contracting leptospirosis is eight times higher in rural populations within the state compared to the urban populations due to various environmental factors and agriculture practices. 

The disease is caused by pathogenic *Leptospira* bacterial species, specifically *Leptospira interrogans,* which has approximately 250 serovars grouped into 24–25 antigenically related serogroups [8,9]. Each serogroup is generally thought to be adapted to one or more animal hosts; dogs, for example, are reservoir hosts for serogroup Canicola, pigs for Bratislava and Pomona, cattle for Hardjo, and certain rodent species for serogroup Icterohaemorrhagiae [2]. Generally, animals that are natural hosts for certain serogroups show no or very limited clinical signs. However, incidental animal hosts infected with different serogroups can lead to severe disease. Ruminants, swine, and equines usually have symptoms characterized by abortions and stillbirths leading to substantial losses in the agricultural sector [10]. 

Human infections occur through direct contact with urine of infected animals such as rodents, livestock, and domesticated pets, and exposure to contaminated objects or the environment such as through soil or water [2]. *Leptospira* can be maintained in wet environments for weeks, although the main sources of the bacteria are domestic and wild mammals that routinely shed specific *Leptospira* serogroups in the urine, allowing for the bacterial persistence in the environment [8,11]. 

Strong surveillance systems are a key strategy for disease prevention, especially for early detection, identification of priority areas, identification of region-specific serovars, and implementation of control efforts after adverse weather events such as flooding, natural disasters, or heavy rain. In Brazil, leptospirosis is a mandatory reportable disease and surveillance activities are implemented around the country [7]. Cases in the country are confirmed through either laboratory testing or the use of epidemiological data alongside clinical symptoms [4]. However, current leptospirosis diagnostic techniques are challenging and complex [5]. The choice of diagnostic test used depends on the phase of the disease; however, most reference laboratories in Brazil use a combination of two serological tests: ELISA IgM screening followed by the microscopic agglutination test (MAT) for testing confirmation [4]. Other direct assays for pathogen detection such as isolation and immunohistochemistry are rarely used in Brazil, while PCR is limited to certain facilities and the National Reference Laboratory. The MAT is considered the gold standard for leptospirosis testing and identifies *Leptospira*-positive samples through the detection of serovar-specific antibodies representing different serogroups [2,4]. However, the test has many limitations including high rates of cross reactivity between serovar specific antibodies, and not all serovars being equally immunogenic, thereby hindering efforts for accurate prediction of infecting serovar [12]. Additionally, persistent low titers in animals can also suggest previous infection or vaccination [13]. The MAT test is still useful however, in providing information about the presumptive infecting serovars circulating in species at the population level [9]. 

Current gaps in detection, surveillance, and response to leptospirosis hinder control programs and the welfare of the communities most severely affected. A better understanding of the factors that affect both the distribution of the various serogroups and potential transmission of the disease by using the One Health approach, will allow to improve prevention and control measures at the local level. This knowledge will provide critical information for decision-makers to be able to target risk areas for priority interventions, prophylaxis treatment, and animal vaccination development.

Furthermore, according to previous studies [9,14], human leptospirosis infections are usually a reflection of the serogroups maintained by the animal population in the region, therefore highlighting the need for serovar-specific vaccine development for human in high-risk populations and areas. Disease mapping and establishing the profile of serogroups circulating is the first step for further understanding both the magnitude and complex epidemiology of the disease, along with its distribution within the study area of Rio Grande do Sul state. The objective of this study was to identify the presumptive infecting *Leptospira* serogroups in human and animal cases and describe their presence by species in the various ecoregions in the state of Rio Grande do Sul, Brazil.

## 2. Materials and Methods 

### 2.1. Study Area

Rio Grande do Sul is the southern-most state in Brazil bordering Uruguay and Argentina to the south and south-west, the Brazilian state of Santa Catarina to the north, and the Atlantic Ocean to the east. The state area is 281,737,888 km^2^ with approximately 10,893,929 inhabitants distributed among 497 municipalities (corresponding to the second subnational administrative level) [15]. The state has one of the highest Human Development Indexes and is primarily agriculturally-driven [15]. 

The state has six distinct ecoregions, with the largest being the Uruguayan savanna spanning the southwest regional border with Argentina and Uruguay, followed by the Parana–Paraiba interior forests, the Araucaria moist forests bordering the state of Santa Catarina to the North, and lastly the Atlantic coast tropical forests and the Serra do Mar coastal forests. There is also a very small ecoregion area bordering Argentina along the Uruguay River called the Mesopotamia, but due to its small size and lack of cases, it was not included in the study [6].

### 2.2. Study Design and Data Collection

This was an eco-epidemiological study with retrospective analysis using secondary data from human and animal passive surveillance systems provided by governmental institutions in Brazil. The study period included the years 2013 to 2015 where human and animal cases were analyzed for the same period. The presumptive serogroups were identified for both human and animal cases and were then aggregated at the second administrative level (municipalities) to describe them by ecoregion. The human leptospirosis cases were gathered from the Brazilian Ministry of Health’s national surveillance database, Sistema de Informação de Agravos de Notificação (SINAN in Portuguese, http://portalsinan.saude.gov.br/). Access to the database was obtained officially from the Pan American Health Organization (PAHO/WHO). This study focused on the confirmed cases in the SINAN database and their laboratory information from the state of Rio Grande do Sul. A descriptive analysis was done using demographic information, occupation, and possible exposure risk factors for human laboratory confirmed cases with MAT.

Animal leptospirosis surveillance data was retrieved from the databased collected from the submitted leptospirosis tests sent to the Laboratory of Animal Research, Instituto de Pesquisas Veterinárias Desideério Finamor (IPVDF). Data quality was reviewed by the local authorities before recorded into the respective database. According to the laboratory records, none of the animals were vaccinated. This database with animal leptospirosis laboratory results was used for a descriptive analysis for the animal species and *Leptospira* serogroups.

### 2.3. Definitions

#### 2.3.1. Confirmed Human Leptospirosis Case Definition

According to the Ministry of Health of Brazil, human cases of leptospirosis present clinical symptoms consistent with the clinical disease and are confirmed by laboratory diagnosis either serologically with ELISA IgM or MAT, or through isolation of the bacteria and detection through PCR [4]. PCR however, is not routinely used and is mainly for a case by case basis as the testing capacity is limited to the National Reference Laboratory [4]. All other laboratory confirmation techniques are available at the state level which is part of the National Public Health Laboratory Network. Additionally, in Brazil, a case can also be confirmed by clinical–epidemiological criteria with selected symptoms with epidemiological history [4].

#### 2.3.2. Confirmed Animal Leptospirosis Case Definition

According to the World Organization for Animal Health (OIE) Terrestrial Manual, a case is confirmed serologically, or via detection of the bacteria through PCR or isolation in conjunction with clinical signs [13]. In endemic areas, serological diagnosis of leptospirosis is confirmed in acute and convalescent samples with a four-fold rise in titer in animals with a compatible clinical illness. In non-endemic areas, however, a single serological sample with a high titer and clinically compatible illness indicates a likely infection [16].

#### 2.3.3. Definitions for Interpreting MAT Results

##### MAT testing for humans:

According to the Brazilian Ministry of Health Leptospirosis Manual [4] for the management and clinical diagnosis of the disease, the serological criteria for case confirmation of suspected cases are one or more of the following:

(1) Reactive ELISA-IgM sample in an acute sample and seroconversion in MAT from an acute sample to a second sample taken 14–60 days after symptom onset, with a titer greater than or equal to 200;
(2) Increase of four times or more in MAT titers between two samples collected within 14 days after the onset of symptoms (maximum of 60 days) between them;
(3) When two or more samples are not available, a single acute sample greater than or equal to a titer of 800 in MAT.

For the purpose of this study, in accordance with the definitions set out by the Brazilian Ministry of Health, confirmed cases in the databases with reported titers of 1:200 or higher in paired samples, or when not available, a titer greater of 800 in single samples were considered for analysis.

##### MAT testing for animals:

According to the World Organization for Animal Health (OIE) Terrestrial Manual, a titer of 1:100 is taken as a positive for the purposes of international trade [13]. Originally developed by Cole et al. 1973 [17], this criterion is still used today as evidenced of prior infection by the recent study by Sathiyamoorthy et al. 2017 [12].

##### Presumptive serogroup MAT definition:

Due to the limitations of MAT for serologic classification of serovars and serogroups, attributed to cross reactions between serovars, for the purpose of this manuscript, the presumptive infecting serogroup was established based on the serogroup with a titer at least two dilutions higher than any other titer in the MAT panel, as previously described by Lelu et al. 2015 [18]. If serogroups were reported with the same titer and none were at least two dilutions higher than the other, then the presumptive serogroup would be classified as undetermined and the samples considered “co-agglutinins” [14].

The MAT panel used by the Ministry of Health of Brazil surveillance system and also the used by the state government of Rio Grande do Sul Agriculture Laboratory, may represent up to 22 serogroups.

#### 2.3.4. Ecoregion Definition

The FAO Terrestrial Ecoregions of the World is a map with a bio-geographic regionalization of the Earth’s terrestrial biodiversity. The bio-geographic units considered as ecoregions, are defined as relatively large units of land or water containing a distinct assemblage of natural communities sharing a large majority of species, dynamics, and environmental conditions [19].

### 2.4. Data Analysis and Management

**Figure A1:**
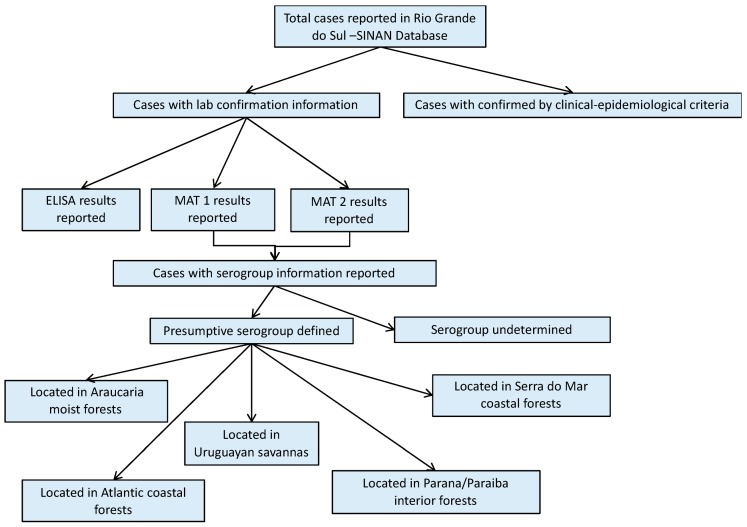
Study design flowchart of human cases from the SINAN database.

**Figure A2:**
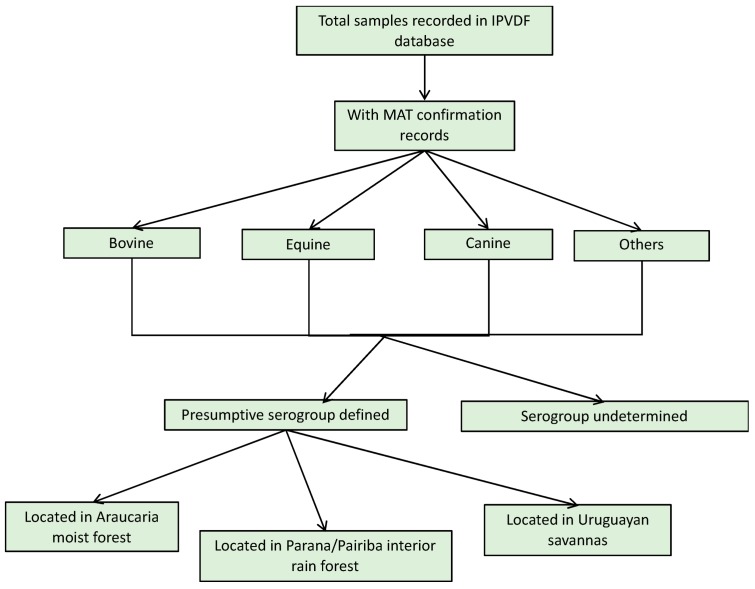
Study design flowchart of animal cases from IPVDF database.

All reported cases (human and animals) in the respective passive surveillance databases were initially included in a combined dataset; however, only confirmed cases with reported *Leptospira* serogroup information were included in the analysis. A flow chart with a description of laboratory testing confirmation and the distribution of leptospirosis cases by ecoregion is provided in the appendix (Figure A1; Figure A2).

The cases (all species) with MAT serovar results were then grouped to the respective serogroups according the definition for presumptive infecting serogroup used in this study [8,18]. A descriptive analysis of the cases with laboratory testing confirmation, serogroups, and the distribution of leptospirosis cases by ecoregion and by municipality was performed.

Data from human cases with the presumptive infecting serogroup identified were summarized with a descriptive analysis of pre-identified risk factors by the Ministry of Health of Brazil and reported demographic characteristics.

Tableau 10.4 and Microsoft Excel 2016 were used for data management and preliminary data visualization. R was used for chi-squared analyses and ArcGIS v10.4 was used to spatially analyze the geographic distribution of the serogroups for both human and animal cases by species at the municipality level and a thematic mapping of ecoregion within the state. The predominant ecoregions by municipality were obtained from a previous study developed in the same state [6,19]. According to the authors of that study, using ArcGIS, zonal statistics by municipality (min, mean, max, standard deviation, range) were calculated for the altitude, slope, temperature, and rain variables. These environmental features were then geo-processed for geometric intersections and shaped the municipal surface of the ecoregions.

## 3. Results

**Figure 1:**
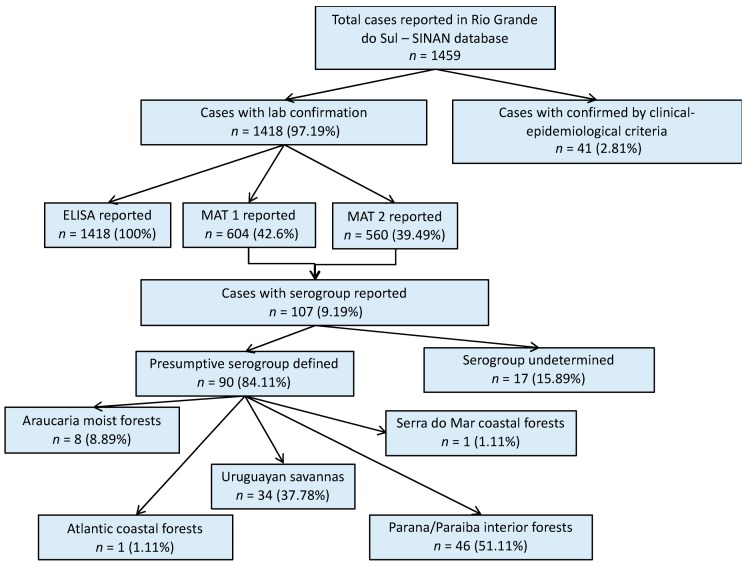
Result flowchart of leptospirosis cases in humans, Rio Grande do Sul, 2013–2015. MAT 1: number of cases with reported results from a single MAT test. MAT 2: number of cases that had reported results from a second MAT test. MAT: microscopic agglutination test.

A total of 1459 human leptospirosis cases were reported in the SINAN database for Rio Grande do Sul within the study period; 97% of them with laboratory confirmation (Figure 1). Among all leptospirosis cases in the SINAN database, ELISA results were reported in 1418 cases (97.12% of all total cases). MAT results were reported in 604 (41.39%) cases, of which 560 had the second MAT test results also reported (MAT 2). Serovar information was available for only 107 (9.19%) single or paired sample results. For 90 of these cases, it was possible to define the presumptive infecting serogroup using the methodology previously described, and then group the cases according to their geographical location within each ecoregion (Figure 1). Cases with the reported presumptive infecting serogroup, were primarily from the Parana–Paraiba ecoregion (51.1%), followed by the Savanah (37.8%).

**Figure A3:**
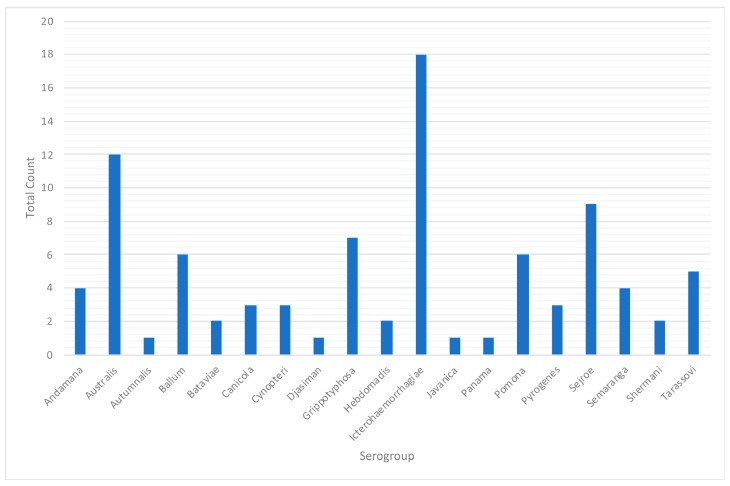
Presumptive infecting serogroups in human leptospirosis cases, Rio Grande do Sul, 2013–2015.

A total of 19 different presumptive infecting serogroups for human cases across the state were identified. Serogroup Icterohaemorrhagiae was the most prevalent (*n* = 18), followed by Australis (*n* = 12) and Sejroe (*n* = 9) (Table 1, Figure A3). Among the cases with the presumptive serogroups identified, in 78% of cases the occupation was related to agriculture; 95.56% reported exposure to local rodents and 72.22% to animal breeding (Table 2). A summary of the demographic characteristics can be found in Table 2 and Table A1. In Table A1, a chi-squared test for independence was performed but no statistical differences were observed among the years.

**Figure 2:**
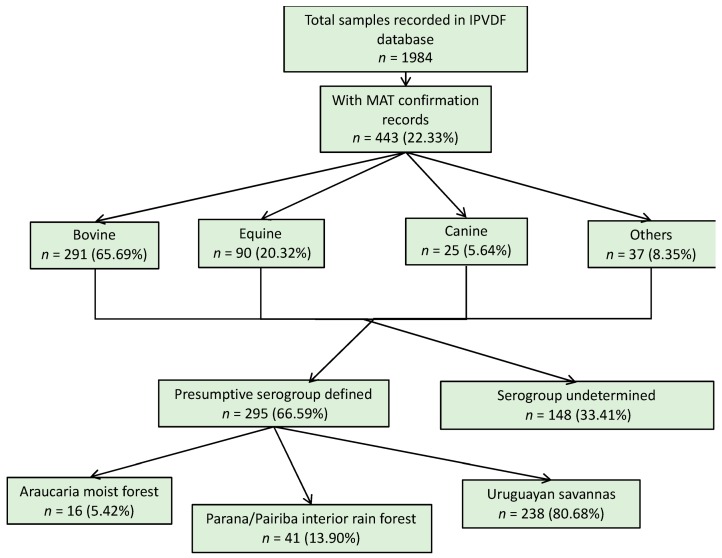
Result flowchart of leptospirosis cases in animals, Rio Grande do Sul, 2013–2015.

**Figure A4:**
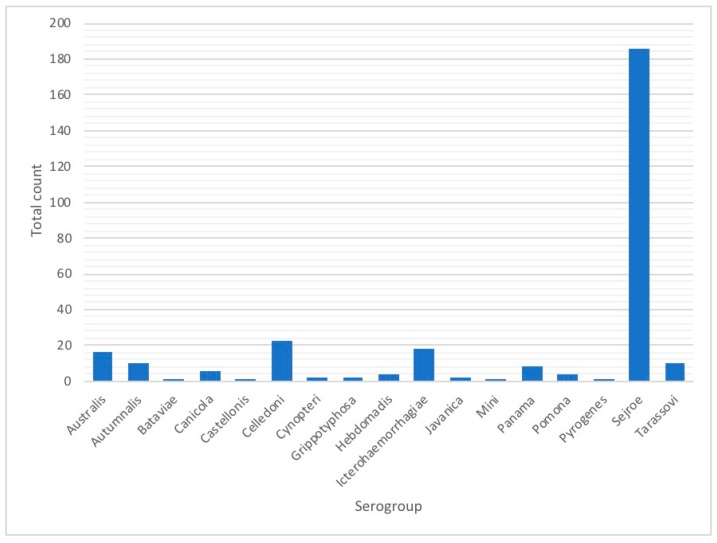
Presumptive infecting serogroups in animal leptospirosis cases, Rio Grande do Sul, 2013–2015.

For the animal species, a total of 1984 samples were reported in the database from IPVDF, of which 443 had MAT testing results and for 295 it was possible to identify a presumptive infective serogroup (Figure 2). The main species were bovine (65.69%), equine (20.32%), and canine (5.64%) (Figure 2). Less common animal species such as ovine (*n* = 26), swine (*n* = 10), and boar (*n* = 1) cases were grouped into a new category called “other”. Within the various animal species, a total of 17 different presumptive infecting serogroups were identified, with the predominant serogroup being Sejroe for 63% of the total animal cases (*n* = 186) followed by Celledoni (*n* = 23) and Icterohaemorrhagiae (*n* = 18) (Table 1; Figure A4).

**Figure 3:**
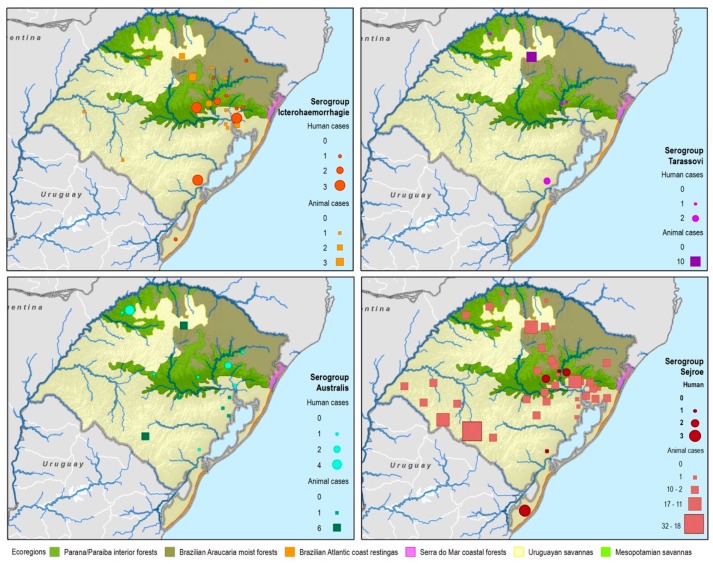
Main presumptive infecting serogroups detected in Rio Grande do Sul in both human and animal cases, 2013–2015.

**Figure 4:**
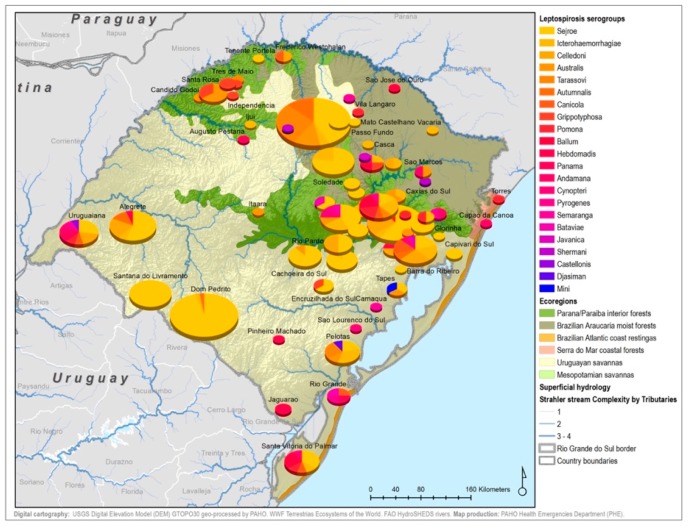
Leptospirosis presumptive infecting serogroup distribution for both human and animal cases within the ecoregions of Rio Grande do Sul, Brazil.

By considering each serogroup separately, regardless of the species and ecoregions, the first five predominant serogroups were Sejroe (50.6%), Icterohaemorrahagiae (10%), Australis (7.2%), Celledoni (6%), and Tarassovi (4%) (Table 1; Figure 3). Of the 497 municipalities within the state, 80 (16.13%) had leptospirosis cases with serogroup information within both databases, with 22 different serogroups in total among seven species (human and six animals). Within those 80 municipalities, only six municipalities had both animal and human cases. The majority were found in the Savannah ecoregion; and in two municipalities, the same presumptive infecting serogroup, Sejroe, was found in humans and animals (Figure 4).

Serogroup information at the municipality level was dispersed among the five ecoregions with a variety of serogroups present in each ecoregion. The Uruguayan savannah and the Parana–Paraiba had the highest number of cases available for determining the presumptive serogroup. Figure 4 shows the spatial distribution by municipalities and species of the serogroups overlaid on top of the ecoregions.

**Figure A5:**
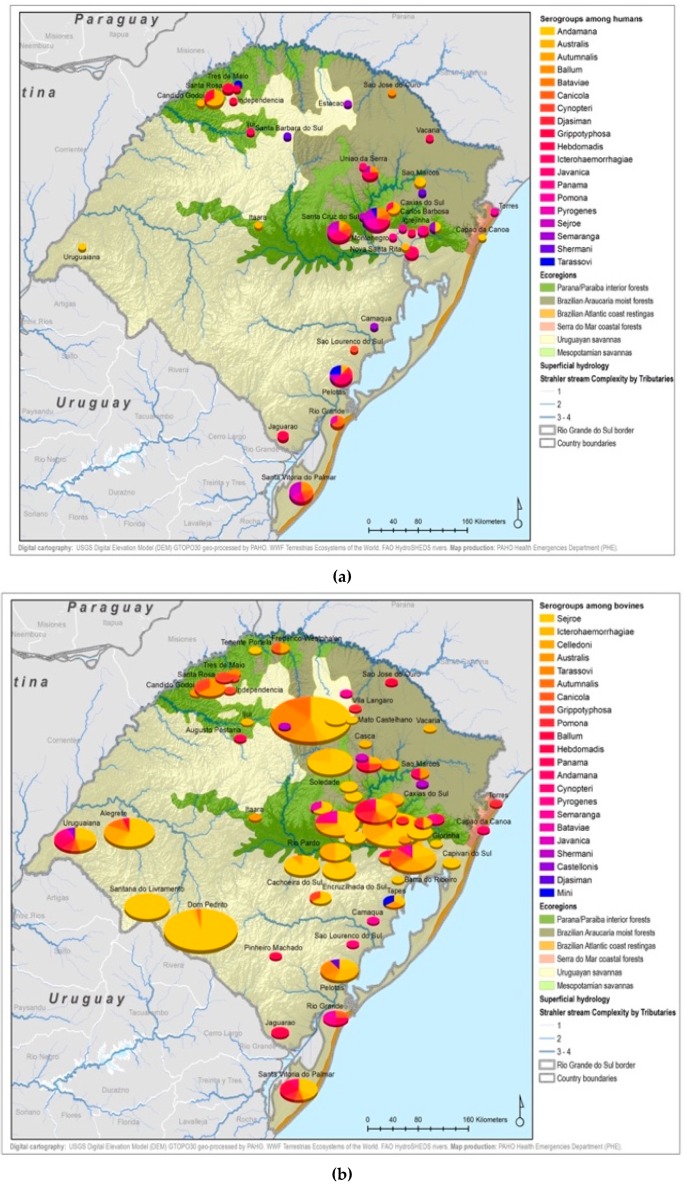

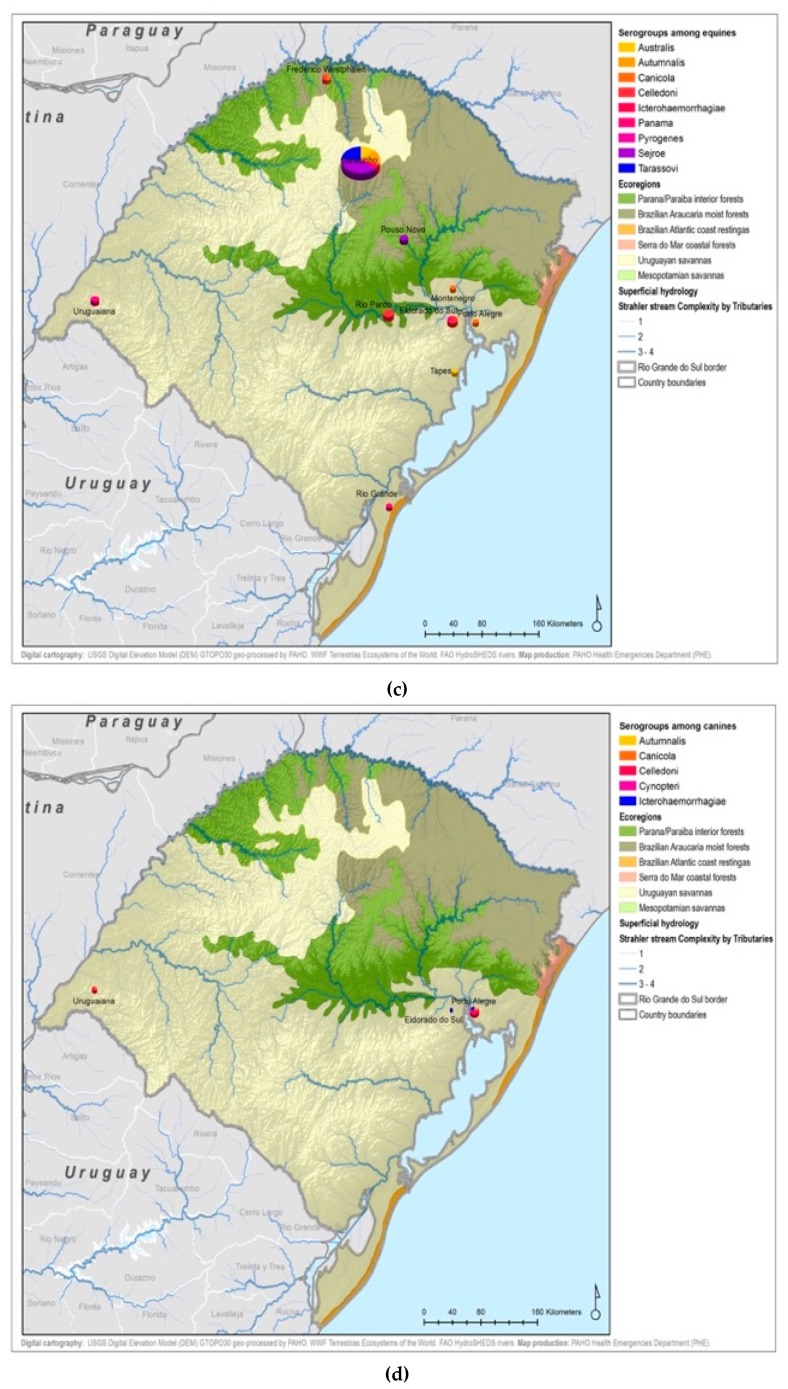

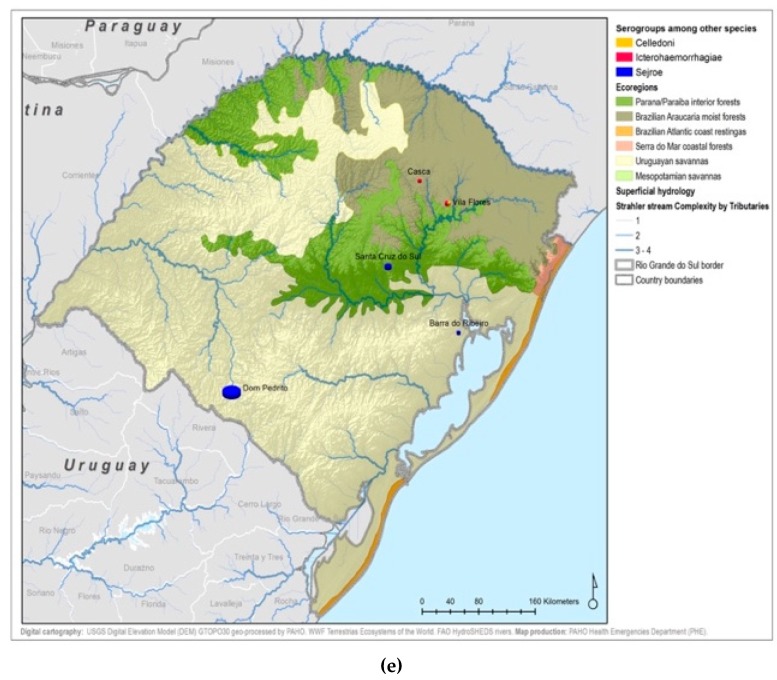
Leptospirosis presumptive infecting serogroups distribution by species within the ecoregions of Rio Grande do Sul in humans (**a**), bovines (**b**), equine (**c**), canines (**d**), others* (**e**) *others: ovine, swine, boar.

**Figure A6:**
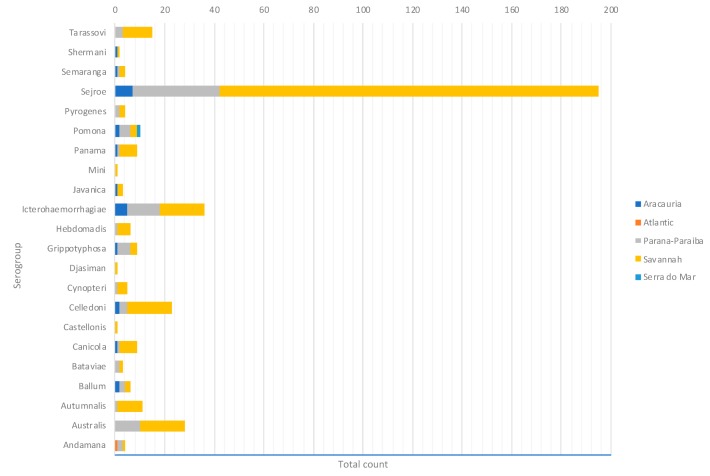
Presumptive infecting serogroups by ecoregion.

All 22 serogroups identifiable by the MAT panel used by the state authorities were reported. Serogroup Sejroe (56.25%) was the most common within the Uruguayan Savanna region. The Parana–Paraiba interior forests also had Sejroe as the predominant serogroup (40.23%), followed by Icterohaemorrhagiae (14.94%). The serogroup Sejroe in bovines was the most present in the Savannah ecoregion, a primarily agricultural-based area. In the Araucaria moist forests, Icterohaemorrhagiae was the dominant serogroup (20.83%), while the Atlantic coast tropical forests and Serra do Mar coastal forests each had limited cases (Table 3; Figure A5; Figure A6). Most of the human cases were distributed between Parana–Paraiba (*n* = 46) and Savannah (*n* = 34) ecoregions, while bovine, canine, and equine cases were predominately located in the Savannah ecoregion 76%, 100%, and 89%, respectively. Additionally, a chi-squared test for independence was performed to observe the relationship between species and ecoregions, and serogroup and ecoregions; in both cases the distributions were not statistically independent (*p* < 0.001).

## 4. Discussion

This spatial description of serogroups circulating in humans and animals within ecoregions allowed for further understanding of the complex epidemiology of leptospirosis in the state of Rio Grande do Sul. Leptospirosis is an excellent example of a disease that does not operate in silos of human medicine, veterinary medicine, and environmental health, but rather constantly mixing between them. Control and prevention efforts need to be multidisciplinary and multi-sectorial, making it a prime candidate for the One Health approach [20].

The findings from this study indicate a large variety of *Leptospira* presumptive infecting serogroups circulating within the state ecosystems among humans and in animal species. This is the first report of serogroup prevalence within the entire state of Rio Grande do Sul associating human and animal passive surveillance data. This investigation provides an important initial epidemiological step in attempting to understand the locations and infection patterns of the various serogroups for this neglected disease. Previous studies have identified the importance of serogroups Canicola and Icterohaemorrhagiae for livestock production in the state, along with serogroups Australis, Autumnalis, Bratislava, Copenhageni, Grippotyphosa, Pyrogenes, and Tarassovi for bovines [21,22]. Other studies focusing on animal production in the southeast area of Rio Grande do Sul also identified a large variety of serogroups present [23]. In terms of human public health, a seroprevalence study focusing on wild, domestic animals, and humans in the coastal area of the state demonstrated the importance of serogroup Icterohaemorrhagiae in the area [21]. These studies highlighted that the serogroups observed, present an important indicator of environmental health due to the diversity of reservoirs in the state, and as a result, recommended improving sanitary practice on farms [21].

By using an eco-epidemiological approach, it was possible to look at various levels of the data (serogroups, cases in different species, municipalities, and ecoregions) in order to better understand the overall context of the disease within the state. The distribution of the same serogroup in the different host animal species and in humans suggests that the strains mentioned are circulating within the ecoregion. However, due to the nature of the surveillance systems and the design of this study, modes of transmission between animal and human cases could not be directly inferred. Furthermore, cross reactivity from the MAT test and different ecologies of animal species and reservoirs in each ecoregion further contribute to the complex epidemiology of leptospirosis. Additional studies specifically addressing the seroepidemiology of leptospirosis, such as one developed in Chile [18], are needed to further the understanding of leptospirosis transmission among species in the state of Rio Grande do Sul.

The information from this study provides a preliminary idea of the distribution of leptospirosis serogroups within the state’s municipalities. The serogroups are also consistent with previous findings regarding the various serogroups and respective primary animal reservoir hosts. Analyzing this data over the ecoregions and comparing it with the land use and agriculture practices, could offer insight into possible transmission sources, and therefore provide better recommendations for the prevention of this disease. Corn, for example, is the primary feed for livestock and is generally stored on the rural properties, attracting rodents to the area. Rodent species that are reservoir hosts and infected with serogroup Icterohaemorrhagiae, could urinate on the feed and contaminate it with *Leptospira.* Additionally, the Savannah ecoregion along the Uruguayan border is primarily focused on cattle and rice production in the grassy plains to the southwest [6]; within this ecoregion, serogroup Sejroe was the predominant serogroup—an important cause of bovine leptospirosis associated with abortions. The other serogroups found in cattle, including Icterohaemorrhagiae and Celledoni, could be likely attributed to accidental infections carried by other animals, farm management, and agricultural practices [21].

In the Parana–Paraiba ecoregion, where the majority of the human cases were located, the predominant serogroup was Icterohaemorrhagiae. This ecoregion is primarily driven by smaller farming communities with fewer livestock, tobacco plantations, and includes urban settlements including the state capital of Porto Alegre [6]. In previous studies, this area was considered a critical risk factor for leptospirosis human cases due to possible environmental persistence of the bacteria in the soil, as studies show it to prefer a pH of 6.5–6.8, also required for certain productive processes when growing tobacco [6,24]. Additionally, the urban areas and larger cities within the Parana–Paraiba ecoregion had the highest number of human cases with serogroup Icterohaemorrhagiae, suggesting increased possible human interactions with rodent populations as potential sources of infection. As most human cases were reported to be in agricultural workers and with reported exposure to rodents, this suggests that they were possibly exposed to a possible rodent reservoir or a contaminated environment (Table 2). These findings are overall in agreement with previous research regarding the prevalence of certain serogroups in infections due to interactions among rodents, humans, domestic ruminants, and other wildlife hosts [9].

In Rio Grande do Sul where the risk of leptospirosis in rural areas is eight times higher than urban areas, preventive activities with the agriculture and animal sectors need to be strengthened [6]. Previous preventive measures have been focused on increasing public awareness of exposure risk when dealing with animals; but through understanding the diversity of leptospirosis serogroups in animals and humans using a One Health approach [25], these findings can help guide public health policy to implement appropriate preventive measures and reduce the impact of leptospirosis. Through spatial analysis, we also conclude in accordance with previous studies that leptospirosis is endemic within the state, and the epidemiology of the disease is further complicated by the high serogroup diversity [6,18,26].

Vaccination is the primary control method for leptospirosis in animal production, specifically targeting decreasing the risk of abortion and negative effects on the reproduction of livestock. As previously stated, the vaccine does not prevent renal shedding of the pathogen, thus allowing for environmental contamination and further spread among herds and onto other wildlife or domestic animals. The commercial leptospirosis vaccines available in Brazil for the immunization of bovines and swine are polyvalent, containing five or more serovars, including Pomona, Icterohaemorrahagiae, Hardjo, Canicola, Wolffi, Grippotyphosa, Bratislava, and Tarassovi [27]. These vaccines are grouped into providing protection against the serogroups Pomona, Icterohaemorrhagiae, Sejroe, Canicola, Tarassovi, Grippotyphosa, and Australis. However, not every vaccine contains every serogroup or specific strain, so understanding the distribution of currently circulating serogroups, chemoprophylaxis, and possible vaccine design and distribution could increase the awareness and preventative efforts in both animal and at-risk human populations.

As such, proper preventative methods need to be established and maintained, and public education needs to continue to further public health interventions and management of the disease. In the warm temperatures of tropical Brazil, correct personal protective equipment are not always worn by those at risk. Therefore, to supplement public health interventions to reduce the number of cases, the use and further development of human vaccines for high-risk populations is important. There are two vaccines licensed for human use developed in Cuba and France; however, the vaccine consists of anti-lipopolysaccharide antibodies that produce a short-term immunity [28]. Furthermore, the immunity is serovar-specific, and the multivalent serovars currently in the two licensed vaccines may not be appropriate for the epidemiological situation in this state [5,28]. Additionally, this study reported the importance of the serogroups Sejroe, Icterohaemorrhagiae, and Celledoni to the bovine population in the state. Of the three mentioned, Celledoni is not included in the commercial animal vaccine licensed by Ministry of Agriculture in Brazil.

Recommendations to the state government also include suggestions to strengthen surveillance and local capacity for case detection through improvement of rapid diagnostic tools available while considering the specific epidemiology of the disease, cost of public health laboratories, and validation of reliable methods. A great limitation arises from the MAT diagnostic test itself, which is currently the gold standard used to determine the presumptive infecting serovar. The MAT test cannot consistently correctly identify the infecting serovar due to the high level of cross reactivity between the antibodies among serovars and antibodies from acute illness, past exposure, and vaccination. Therefore, identifying infecting serovars solely through MAT serology in both human and animal sera limits the accuracy of the results. However, results from the MAT test should still be indicative of the circulating serogroups within the specific geographical area for each species at the population level [9]. Nevertheless, further research and development is needed for more accurate diagnostic tools for leptospirosis and possible incorporation of PCR for case confirmation. The use of culture to accurately serotype isolates is difficult to implement routinely and requires material to be sent to a reference laboratory for diagnosis and identification of the clinical isolates. Clinically, the finding of specific serogroups does not alter or affect course of treatment or outcome; however, health personnel still need to be trained in proper case management and detection, and collaboration needs to be promoted with the reference laboratories for diagnostic testing.

This research promotes state and government recommendations to strengthen surveillance and local capacity for case detection through improvement of rapid diagnostic tools available for both human and animal infections. In addition to increasing patient management, health promotion, and education, stronger preventative and protection methods are needed to support public health. Finally, these findings also address a key aspect of the Global Leptospirosis Environmental Action Network (GLEAN) priorities for research, technological development, and innovation (RTDI), specifically the biodiversity and geographic distribution of *Leptospira spp*. and its serovars [5]. By highlighting the serogroup distributions across humans and animals within the environment, understanding of this neglected zoonotic disease can be increased. In turn, this will contribute to research and health policies based on country needs. Further epidemiological studies and possible prediction models using the SINAN database can be considered along with further seroepidemiological surveys in domestic animals and livestock at each ecoregion to better understand possible transmission routes and environmental persistence. It is imperative to continue a holistic and One Health approach, considering human, animal, and ecosystem interactions to assess the complex epidemiology of leptospirosis which has been shown to play a key role in disease persistence and transmission.

## Figures and Tables

**Table 1:** Leptospirosis presumptive infecting serogroups by species, Rio Grande do Sul, 2013–2015.

Serogroup	Species					
	*n* (% Serogroup Total)					Total *n* (% Total)
	Human	Bovine	Canine	Equine	Others	
Andamana	4 (100)	-	-	-	-	4 (1.04)
Australis	12 (42.86)	7 (25.00)	1 (3.57)	8 (28.57)	-	28 (7.27)
Autumnalis	1 (9.09)	3 (27.27)	1 (9.09)	6 (54.55)	-	11 (2.86)
Ballum	6 (100)	-	-	-	-	6 (1.56)
Bataviae	2 (66.67)	1 (33.33)	-	-	-	3 (0.78)
Canicola	3 (33.33)	-	3 (33.33)	3 (33.33)	-	9 (2.34)
Castellonis	-	1 (100)	-	-	-	1 (0.26)
Celledoni	-	9 (39.13)	6 (26.09)	7 (30.43)	1 (4.35)	23 (5.97)
Cynopteri	3 (60.00)	-	2 (40.00)	-	-	5 (1.30)
Djasiman	1 (100)	-	-	-	-	1 (0.26)
Grippotyphosa	7 (77.78)	2 (22.22)	-	-	-	9 (2.34)
Hebdomadis	2 (33.33)	4 (66.67)	-	-	-	6 (1.56)
Icterohaemorrhagiae	18 (50.00)	10 (27.78)	4 (11.11)	2 (5.56)	2 (5.56)	36 (9.35)
Javanica	1 (33.33)	1 (33.33)	-	1 (33.33)	-	3 (0.78)
Mini	-	1(100)	-	-	-	1 (0.26)
Panama	1 (11.11)	2 (22.22)	2 (22.22)	4 (44.44)	-	9 (2.34)
Pomona	6 (60.00)	4 (40.00)	-	-	-	10 (2.60)
Pyrogenes	3 (75.00)	-	-	1 (25.00)	-	4 (1.04)
Sejroe	9 (4.62)	142 (72.82)	-	19 (9.74)	25 (12.82)	195 (50.65)
Semaranga	4 (100)	-	-	-	-	4 (1.04)
Shermani	2 (100)	-	-	-	-	2 (0.52)
Tarassovi	5 (33.33)	-	-	10 (66.66)	-	15 (3.90)
TOTAL	90	187	19	61	28	385

**Table 2:** Occupation and possible risk factors for human leptospirosis cases, Rio Grande do Sul, 2013–2015.

Variable	*N* (Total %)
**Occupation**	***n* = occupation**
Livestock farmer	2 (2.22)
Mason	3 (3.33)
Agriculture producer	3 (3.33)
Rice farmer	4 (4.44)
Part-time agricultural hire	5 (5.56)
Truck driver	6 (6.67)
Agricultural worker	12 (13.33)
Not mentioned	55 (61.11)
**Reported exposure risk factors**	***n* = risk Factors**
Water tank	7 (7.78)
Sewage	12 (13.33)
Direct contact rodents	27 (30.00)
Water, mud, flooding	31 (34.44)
Rubbish/rubble	32 (35.56)
Proximity to river/stream/dam	36 (40.00)
Grain/food storage	37 (41.11)
Wasteland	41 (45.56)
Planting/harvesting	49 (54.44)
Animal breeding	65 (72.22)
Local rodents	86 (95.56)

**Table 3:** Leptospirosis presumptive infecting serogroups and species by ecoregions.

Variable	Ecoregions					
	*n* (% Species by Ecoregion)					
	Araucaria	Atlantic	Parana-Paraiba	Savannah	Serra do Mar	Total
**Species**						
Human	8 (33.33)	1 (100)	46 (52.87)	34 (12.5)	1 (100)	90 (23.38)
Bovine	11 (45.83)	-	33 (37.93)	143 (52.57)	-	187 (48.57)
Equine	2 (8.33)	-	5 (5.75)	54 (19.85)	-	61 (15.84)
Canine	-	-	-	19 (6.99)	-	19 (4.94)
Others	3 (12.50)	-	3 (3.45)	22 (8.09)	-	28 (7.27)
**Serogroups**						
Andamana	-	1 (100)	2 (2.30)	1 (0.37)	-	4 (1.04)
Australis	-	-	10 (11.49)	18 (6.12)	-	28 (7.27)
Autumnalis	-	-	1 (1.15)	10 (3.68)	-	11 (2.86)
Ballum	2 (8.33)	-	2 (2.30)	2 (0.74)	-	6 (1.56)
Bataviae	-	-	2 (2.30)	1 (0.37)	-	3 (0.78)
Canicola	1 (4.17)	-	1 (1.15)	7 (2.57)	-	9 (2.34)
Castellonis	-	-	-	1 (0.37)	-	1 (0.26)
Celledoni	2 (8.33)	-	3 (3.45)	18 (6.62)	-	23 (5.97)
Cynopteri	-	-	1 (1.15)	4 (1.47)	-	5 (1.30)
Djasiman	-	-	-	1 (0.37)	-	1 (0.26)
Grippotyphosa	1 (4.17)	-	5 (5.75)	3 (1.10)	-	9 (2.34)
Hebdomadis	-	-	1 (1.15)	5 (1.84)	-	6 (1.56)
Icterohaemorrhagiae	5 (20.83)	-	13 (14.94)	18 (6.62)	-	36 (9.35)
Javanica	1 (4.17)	-	-	2 (0.74)	-	3 (0.78)
Mini	-	-	-	1 (0.37)	-	1 (0.26)
Panama	1 (4.17)	-	1 (1.15)	7 (2.57)	-	9 (2.34)
Pomona	2 (8.33)	-	4 (4.60)	3 (1.10)	1 (100)	10 (2.60)
Pyrogenes	-	-	2 (2.30)	2 (0.74)	-	4 (1.04)
Sejroe	7 (29.17)	-	35 (40.23)	153 (56.25)	-	195 (50.65)
Semaranga	1 (4.17)	-	1 (1.15)	2 (0.74)	-	4 (1.04)
Shermani	1 (4.17)	-	-	1 (0.37)	-	2 (0.52)
Tarassovi	-	-	3 (3.45)	12 (4.41)	-	15 (3.90)
TOTAL	24	1	87	272	1	385

* Species chi-squared of 87.942 *p* < 0.001 and for serogroup chi-squared of 446.88 *p* < 0.001.

## Appendix A

**Table A1:** Demographic summary of human cases. Sex chi-squared 0.54515, p-value = 0.7614; race chi-squared 6.8153, p-value = 0.146; and human-settlement chi-squared 2.274, *p*-value = 0.6855.

Variable	2013 *n* (% Total)	2014 *n* (% Total)	2015 *n* (% Total)	TOTAL *n* (% Total)
**Sex**				
Female	2 (2.22)	4 (4.44)	5 (5.56)	**11 (12.22)**
Male	11 (12.22)	38 (42.22)	30 (33.33)	**79 (87.78)**
**Race**				
Black	2 (2.22)	-	2 (2.22)	**4 (4.44)**
Parda	-	1 (1.11)	-	**1 (1.11)**
White	11 (12.22)	41 (45.56)	33 (36.67)	**85 (94.44)**
**Human settlement**				
Urban	10 (11.11)	25 (27.78)	23 (25.56)	**58 (64.44)**
Rural	3 (3.33)	16 (17.78)	12 (13.33)	**31(34.44)**
Peri-urban	-	1 (1.11)	-	**1 (1.11)**
